# Consumption of vitamin A rich foods and its associated factors among children aged 6–59 months in North Shoa Zone, Oromia regional state, Ethiopia

**DOI:** 10.3389/fnut.2025.1526292

**Published:** 2025-06-16

**Authors:** Meseret Moroda, Girma Garedew Goyomsa, Rebik Shukure, Shelema Nigussu

**Affiliations:** ^1^Department of Public Health, Salale University, Fitche, Ethiopia; ^2^Department of Horticulture, Salale University, Fitche, Ethiopia

**Keywords:** consumption of vitamin A rich foods, children aged 6–59 months, associated factors, North Shoa Zone, Ethiopia

## Abstract

**Background:**

Vitamin A deficiency is a major nutritional concern in poor societies, especially in lower income countries. In Ethiopia vitamin A deficiency was high in preschool children. Recently, the government of Ethiopia has been strengthening nutrition-sensitive programs that focus on promoting healthy dietary practices as a public health intervention to manage vitamin A deficiency. However, there is limited research on the consumption of vitamin A-rich foods among children aged 6–59 months in the study area.

**Objectives:**

To assess magnitude of consumption of vitamin A rich foods and its associated factors among children aged 6–59 months in North Shoa Zone, Oromia, Ethiopia, 2022.

**Methods:**

Community based cross sectional study was conducted at North Shoa Zone, Ethiopia in 2022. A total of 916 mothers’ pair children of age 6–59 months were included. A multistage stratified sampling followed by the simple random sampling technique was used to select the study participants. Data was collected through a face to face interview. STATA version 14 was used for analysis. Adjusted Odds Ratio (AOR) with a 95% CI and a *P*-values < 0.05 was used to declare statistical significance in the multivariable analysis.

**Results:**

Overall, magnitude of Consumption of vitamin A rich food of children aged 6–59 months was 39.1% (95% CI: 37.71, 40.49) in a week before survey. In multivariable regression analysis, maternal educational levels above secondary education and above (AOR = 1.81, 95% CI;1.19, 2.75), paternal educational levels above secondary education and above (AOR = 1.54, 95% CI: 1.05, 2.23),house hold income 3,000 and above ET birr (AOR = 2.18, 95% CI: 1.24, 3.82),adequate dietary diversity score (AOR = 1.48, 95% CI: 1.05, 2.07),adequate meal frequency (AOR = 1.37, 95% CI: 1.04, 1.81) and had nutrition counseling (AOR = 1.31, 95% CI: 1.10, 1.73) were factors positively increases vitamin A consumption among children aged 6–59 moths, Whereas, age of children between 11 and 17 months aged 0.56 (0.33, 0.90) and 18–23 months aged 0.46 (0.29, 0.74) months was negatively associated.

**Conclusion:**

This study results showed that vitamin A consumption children aged 6–59 months was low. Strategies to increase vitamin A consumption should focus on promoting parental education, enhancing dietary diversity and meal frequency, supporting household income generation, targeting age groups in the transition to complementary feeding and strengthening nutrition counseling on child feeding practices.

## Introduction

Vitamin A is an essential micronutrient crucial for eye function and immune system regulation (1). Infants, toddlers under five, and pregnant or breastfeeding mothers are at the highest risk of vitamin A deficiency (VAD) (2). Preschool children are particularly vulnerable due to their high vitamin A requirements relative to body weight and increased susceptibility to infections (2).

Vitamin A–rich foods are generally categorized into two types: preformed vitamin A from animal sources and provitamin A carotenoids from plant sources. Animal-based sources, which provide the active form of vitamin A that the body can readily use, include liver (from beef, chicken, or goat), egg yolks, butter, cheese, whole milk, and fish liver oils such as cod liver oil. On the other hand, plant-based sources contain provitamin A carotenoids, which the body converts into vitamin A. These include carrots, sweet potatoes, pumpkins, mangoes, papayas, dark leafy greens such as spinach, kale, collard greens, and amaranth leaves, as well as red palm oil and red or orange-fleshed fruits and vegetables like cantaloupe and red bell peppers (3).

Globally, VAD remains a significant public health concern, affecting 33.3% of preschool-aged children (3). The burden is higher in low-income regions such as sub-Saharan Africa (48%) and South Asia (44%) (3). In Ethiopia, VAD prevalence among preschool children is estimated at 61%, posing a serious nutritional challenge (4). VAD is associated with protein-energy malnutrition (PEM), leading to night blindness, corneal damage, and increased child mortality. It is estimated to contribute to over 100 million cases worldwide, accounting for one in four child deaths in affected regions. Improving vitamin A intake can reduce child mortality by 23% or more (5).

Vitamin A-rich food consumption plays a crucial role in reducing morbidity and mortality. A meta-analysis of 17 studies reported a 24% reduction in all-cause child mortality with adequate vitamin A intake (6). Furthermore, vitamin A-rich diets and supplementation have been shown to lower diarrhea-related deaths in children by 28% (2).

Key global strategies to combat VAD include exclusive breastfeeding, vitamin A supplementation, dietary diversification, and food fortification (1). In Ethiopia, national nutrition programs have been implemented to promote home gardening, community horticulture, and increased access to animal-source foods (7). The “Seqota” Declaration (2015–2030) further aims to eliminate malnutrition in children under two by 2030. Despite these efforts, VAD remains a public health issue in Ethiopia, causing approximately 80,000 child deaths annually (8). The prevalence of Bitot’s spots (1.46%) and night blindness (1.22%) exceeds WHO public health thresholds (9). Furthermore, a review and meta-analysis revealed a statistically insignificant decrease in Bitot’s spot prevalence from 2.2% to 1.8% between 2005 and 2019. Seven out of eight investigations on subclinical vitamin A deficiency showed a major public health problem (> 20%) (10).

Globally, only 20.8% of children aged 6–24 months consume fruits and vegetables daily, while 40.1% consume vitamin A-rich foods (11). In low- and middle-income countries, only 18% of children meet WHO’s recommended daily vitamin A intake (12). Strategies to address micronutrient deficiencies is increased consumption of fruits and vegetables specifically vitamin A deficiency (3, 13).

In Ethiopia, 62% of children aged 6–23 months do not consume adequate vitamin A-rich foods (14). Studies indicate that only 7%–39% of children consume plant-based vitamin A sources (15–17), while 12%–24% consume animal-source vitamin A foods (15).

However, there is limited research on vitamin A-rich food consumption among preschool children in the North Shoa Zone. Data on the prevalence and determinants of vitamin A-rich food intake in this region are scarce. This study aimed to assess the magnitude of vitamin A-rich food consumption and identify associated factors among children aged 6–59 months. The findings will be valuable for policymakers, program administrators, and researchers working in child nutrition.

## Materials and methods

### Study area and period

The study was conducted at North Shoa Zone, Oromia, Ethiopia from 06 February 2022 to 11 April 2022. Fitche is administrative center of North Shoa which is located 114 km away in the North West from Addis Ababa. According to information obtain from the Fitche Zone administrative office; the zone has 12 woreda and 296 kebeles (lowest local administrative units of the country in the administrative structure of the country). The Zone has five hospitals, 64 health centers and 268 health posts. The number of under five children were 277,733 of this 81,970 were under two age children (16). The major food products are grain crops like Teff, Barley, Wheat, Maize, Sorghum, Oath, Bean, and other legumes. In addition oil seeds like Neug, Linseed, Groundnuts, Safflower, Sesame and others. potato, spinach, lettuce, cabbage, kale, pumpkin, and fruits available at the study area include mango, avocado, banana and orange (17).

### Study design

Community based cross-sectional study was conducted.

### Source and study population

Source population: All children aged 6–59 months with mother/caregiver reside in North Shoa were source population of the study.

Study population: All children aged 6–59 months with mother/caregiver reside in selected kebeles of North Shoa were the study population.

Inclusion criteria: All children 6–59 months with mother/caregiver in North Shoa Zone those resides more than 6 months.

Exclusion criteria: children’s mothers/caregiver those can’t communicate, hear and ill was excluded. Additionally children with chronic disease will be excluded because children with chronic disease are just unique in their preference and eating habits which may under estimate the magnitude of vitamin A consumption.

### Sample size and sampling procedures

#### Sample size determination

The sample size was calculated using a single population proportion formula by considering 95% confidence level, 4% of margin of error and the proportion of good consumption of vitamin A rich foods 38% (14).

*n* = (Z_α 1/2_)^2 *P*(1–*p*)^ = (1.96)^2^ 0.38(0.62) = 566

d^2^/(0.04)^2^

*n* = Minimum sample size for a statistically significant survey.

Z = Normal deviant at the portion of 95% confidence interval two-tailed test = 1.96.

P = proportion of vitamin A rich food consumption = 38%.

q = 1–p.

d = margin of error acceptable is taken as 4% = 0.04.

Finally, 934 sample size was calculated by single population after considering of a design effect of 1.5 and 10% non-response rate.

A multistage stratified sampling followed by systematic random sampling technique was used to select the study participants. Initially, from the 12 woreda in North Shoa zone, four woreda (Wara Jarso, Degam, Yaya Gulale and Wuchale) were selected by simple random sampling using lottery method. Then each woreda was stratified by considering rural and urban as strata. Specifically, Wara Jarso has 25 kebeles, of which one urban and four rular were selected; Degam has 18 kebeles, with one urban and three rular were selected; Yaya Gulale has 17 kebeles, with one urban and three rular were selected; and Wuchale has 24 kebeles, with one urban and three rular were selected. Totally 17 kebeles four urban and 13 rural was randomly selected using random sampling technique. The projected total population size in the selected kebeles is 48,284 of which 4,173 is children 6–59 months of age. List of households with children 6–59 months in the selected kebeles was obtained from health post and woreda administrative. The required number of participants from each kebele was determined based on proportion to population size allocation. Since the selected kebeles had a relatively similar number of households, we combined the total number of eligible households across all selected kebeles and divided it by the total sample size to calculate the sampling interval. Based on this, study participants were selected using a systematic random sampling technique at every 5th interval (K = 4173/934 ≈ 5). The k value was calculated by using total number house hold in selected seventeen kebeles with children aged 6–59 months divided to total sample size. The beginning sample was selected from the first five registers by simple random sampling.

### Study variables

#### Dependent: consumption of vitamin A rich food groups

Independent: Socio-demographic and economic; Mothers age, child age, place of residence, marital status, mothers, educational level, father educational level, mother occupation, child dietary diversity, meal frequency, family size, household income, home gardening, exposure to media.

Nutrition related variables: mother’s knowledge on vitamin A consumption, vitamin A practice, nutrition counseling, had discussed on child feeding, dietary diversity, meal frequency.

### Operational definitions

Adequate Vitamin A-rich food consumed: Consumption of animal sources of vitamin A for > 4 days per week, or consumption of weighted source (total consumption of animal and vegetable sources of vitamin A) for > 6 days per week (18, 19).

Minimum child dietary diversity (MDD): If children received at least five (from eight food groups for 6–23 aged month and for 24–59 months from ten food groups) in the preceding 24 h of the interview considered have adequate DD MDD (20).

Minimum meal frequency: meal frequency of the child was determined by asking the mother how many times the child took solid, semi-solid, or soft foods in the 24 h preceding the survey. Accordingly, two or more times for breastfed infants 6–8 months of age, three or more times for breastfed children 9–23 months, and four times for non-breastfed children 6–23 months were considered as the children received the minimum meal frequency and for 2–5 years four and above per day (20).

Satisfactory exposure to media: Mothers/caregivers of the children at least once a week read a newspaper or magazine or listen to radio, or watched television. Less exposure than once a week was defined as unsatisfactory exposure to media (18).

Mother/care giver knowledge on vitamin A rich food source: was determined using knowledge item questions (eight items) which were adopted from monitoring vitamin A deficiency key indicators. Then, if the mothers correctly answer above the mean of knowledge questions, she was considered as having a good knowledge, otherwise, she has a poor knowledge (21).

Nutrition counseling: During data collection, the participants were asked if they had received child nutrition counseling from providers such as health services centers and home visits by nutritionists or health and agricultural workers in the last 12 months yes/no (22).

Antenatal Care (ANC): Receiving at least one antenatal care visit from a skilled health provider (such as a nurse, midwife, or health officer) during the mother’s most recent pregnancy with the child under 2 years of age. ANC includes counseling or information on maternal and child nutrition, including the importance of consuming vitamin A–rich foods (22).

Postnatal Care (PNC): Health services provided to mothers and their newborns within the first 12 months after delivery, which may include counseling on infant and young child feeding practices (23).

### Data collection tools and quality control

The data will be collected through a face to face interview, using a structured and pre-tested questionnaire. The questionnaire was adopted from EDHS 2016, Hellen Keller International Food Frequency Questionnaire (FFQ) after adapting to the local context for measuring the consumption of vitamin A-rich foods (19) and WHO standardized questionnaire for IYCF practices indicators to measure children dietary diversity (20).

Consumption of vitamin A-rich food was assessed with food items listed in the Hellen Keller guideline adapted to local. The lists of food item included for assessment of vitamin A-rich food consumption include animal sources (fresh cow milk, egg, fish, meat (beef, lamb, chicken),organ meat (liver, kidney, heart),butter) and plant source foods (dark green leafy vegetables (DGLVs), carrot, pumpkin, mango, papaya, red bell pepper) Supplementary Table 1. The FFQ asks respondents how many days in the last week they consumed the foods listed. The questionnaires prepared in English and translated to Afan Oromo and retranslated back to English version to maintain consistency. In order to maintain the quality of data, 2 days training was given to data collectors and supervisors by the principal investigator.

A pre-test was done on 5% of the sample out of the study area. A total of 12 clinical nurse data collectors and four public health expert supervisors were recruited for the study. During the data collection period, a close supervision was done by the principal investigator and the supervisors. The accuracy and completeness of the collected data was checked during and at the end of data collection daily by the principal investigator. Moreover, the data was checked by inspection, and crosschecking the entered data with the questionnaire.

### Data processing and analysis

All of the returned copies of the questionnaire was manually checked for completeness and consistency of responses. Then, double data entry was done using EPI-INFO Version 07 and exported to STATA version 14 further analyses. Descriptive statistics summarized by using figures, tables, and texts.

Factors associated with the consumption of vitamin A-rich food sources was identified by using a binary logistic regression model. A binary logistic regression model fitted after checking the necessary assumptions of the model to identify factors associated with consumption of vitamin A rich foods and Variables with *P*-values of < 0.2 in the bi-variable analysis entered in to the multivariable analysis to control possible effects of confounders. Both Crude Odd Ratio (COR) and Adjusted Odds Ratio (AOR) with a 95% of confidence interval was used to examine the strength of associations, and a *P*-values < 0.05 will be used to declare statistical significance in the multivariable analysis.

### Ethical consideration

Ethical clearance was obtained from Salale University Ethical review board. Official support letter to conduct the study was obtained from North Shoa Zone, Fitche Administration head and respective woreda. Verbal informed consent was taken from each mothers/caregivers of child (study participants) after the study purpose, procedure and duration, possible risks and benefits of the study was clearly explained for the participants using local language. Study participants was told they have full right to participate or not, and they would also informed that all the data obtained from them was be kept confidential using codes instead of any personal identified.

## Results

### Socio-demographic and economic characteristics of mothers’ with children aged 6–59 months

Out of 934 eligible study participants, 916 mothers’ with their children agreed to participate in this study, which made the response rate 98.1%. More than half (53.4%) of the children were female and near one fourth (27%) children were in the age group of 36–59 months. The median age of mothers was 29 years (inter-quartile range18–40 years). Of all mothers, 93.2% were in union of marital status, and 58.1% of mothers had no formal education. Regarding house hold income; about half of the children (45.7%) were from house hold income less than 2500/month Birr and About 55.9% mothers were received nutrition counseling (Table 1).

**TABLE 1:** Socio demographic and economic characteristics of children aged 6–59 months and with their mother at North Shoa Zone, Oromia regional state, Ethiopia, 2022 (*n* = 916).

Variables	Categories	Frequency	Valid percent
Child age (months)	6–15	127	13.9
	16–25	152	16.6
	26–35	169	18.4
	36–45	221	24.1
	46–59	247	27.0
Child sex	Female	489	53.4
	Male	427	46.6
Residence	Urban	151	16.5
	Rural	765	83.5
Age of respondents (in year)	15–24	236	25.8
	25–34	450	49.0
	35–50	200	21.8
Ethnicity	Oromo	799	87.2
	Amara	89	9.7
	Other a*	28	3.1
Religion	Orthodox	774	81.2
	Protestant	104	11.4
	Muslim	56	6.1
	Others b*	12	1.3
Mothers educational level	No formal education	532	58.1
	Primary school	250	27.3
	Secondary school and above	134	14.6
Mother’s occupation	House wife	830	90.6
	Employed	53	5.8
	Others c*	33	3.6
Marital status of mother	In union	854	93.2
	Not in union	62	6.8
Father educational level	No formal education	449	49.0
	Primary school	282	30.8
	Secondary and above	185	20.8
Father occupation	Farmer	594	64.8
	Employed	197	21.5
	Trader	85	9.3
	Other d*	40	4.4
House hold member	< 4	355	38.8
	> 5	561	61.2
House hold income/month	Less than 2,000	314	34.3
	2,500–3,499	326	35.6
	3,500 and above	276	30.1
Media exposure	Yes	251	27.4
	No	665	72.6
Distance of health facility (time take)	Less than 30 min	363	39.6
	Greater or equal to 30 min	553	60.4

*Other a includes Gurage, Tigre, and Woliyita ethnicity, other b includes Catholic, Adventist, and Wakefata religions, other c includes farmers and Merchant maternal occupation, Other d includes daily labor, Self-employed, and teacher fathers occupations.

Near more than half (52.5%) of children had adequate meal frequency per day and 17.2% of children were had adequate dietary diversity (Table 2).

**TABLE 2:** Nutrition related variables of children aged 6–59 months and with their mother at North Shoa Zone, Ethiopia, 2022 (*n* = 916).

Variables	Categories	Frequency	Percent
Exclusive breast feeding (for 6–24 months)	Yes	164	64.3
	No	92	35.7
ANC visit (6–24 months)	Yes	158	61.7
	No	98	39.3
Place of delivery (6–24 months)	Institution	65	25.4
	Home	191	74.6
PNC visit (6–24 months)	Yes	51	19.9
	No	205	80.1
Had nutrition counseling	Yes	512	55.9
	No	404	44.1
Discussed on child feeding	Yes	257	27.5
	No	677	72.5
Meal frequency/day	< 4	481	52.5
	> 4	435	47.5
Dietary diversity	Five and above	158	17.2
	Less than five	758	82.8
Home gardening	Yes	289	32.5
	No	627	67.5
Vitamin A consumption	Adequate	358	39.1
	Not adequate	558	60.9
Mother’s knowledge of vit. A	Good	356	38.9
	Poor	559	61.1
Mother’s practices of vit. A	Good	241	25.8
	Poor	693	74.2
**Food groups of dietary diversity**			
Grains	–	734	78.6
Pulses, beans, peas and lentils	–	348	24.3
Nuts and seeds	–	189	15.2
Milk and milk products	–	542	37.4
Meat, poultry and fish	–	89	5.5
Egg	–	214	19.9
Dark green leafy vegetables	–	198	15.2
Other vitamin A- rich fruits and Vegetables	–	156	16.7
Other vegetables and fruits	–	105	9.3
Oils and fats	–	113	1.9

ANC, Antenatal Care; PNC, Postnatal care; vit.A, vitamin A.

### Magnitude of consumption of foods rich in vitamin A among children aged 6–59 months

**FIGURE 1:**
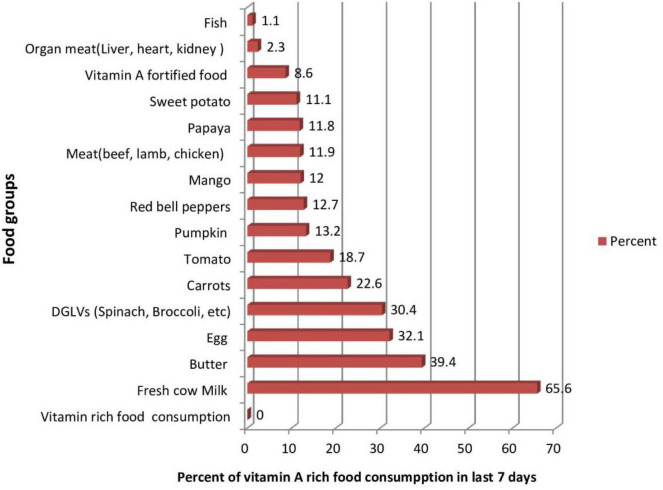
Vitamin A-rich food groups consumed at least once during last week among children aged 6–59 months at North Shoa Zone, Oromia, Ethiopia, 2022 (*n* = 916).

Overall, magnitude of consumption of vitamin A rich food of children aged 6–59 months was 39.1% (95% CI: 37.71, 40.49) in a week before survey in North Shoa Zone. This study revealed that 11.8%–30.4% of children aged 6–59 months consume plant source foods. Whereas animal source food consumption was low as fish consumption 1.1% to high cow milk consumption 65.6% (Figure 1).

Intake of cow milk, butter, eggs, and dark green leafy vegetables were the most commonly consumed food items, with 65.5%, 39.4%, 32.5%, and 30.4% of children consuming them, respectively, whereas meat (beef, pork, lamb, chicken), organ meat (liver, heart, kidney) and fish were the least intake food items which 11.9%, 2.3%, and 1.1% of children intake them, respectively (Figure 1). This study results showed that less than 4% of children aged 6–59 months consumed fruit and vegetable 3–4 servings, and only 2% had five and above serving per day.

### Factors associated with poor consumption of vitamin A-rich food sources among children aged 6–59 months

The association between consumption of vitamin A-rich foods and predictors was analyzed using binary logistic regression. In the bi-variable binary logistic regression analysis, child age, educational level of mother, father educational level, father occupation, number of house hold members, house hold income/month, discussed on child feeding, nutrition counseling, nutrition knowledge, meal frequency and dietary diversity had *p*-value of less than 0.2; accordingly passed the variable screening criteria then fitted to multivariate analysis.

But variables like mother age, occupation of mother, marital status, place of residence, exposure to media, home gardening, nutrition practices, and time taken to health facility were not significant at *p*-value less 0.2; hence, these variables were excluded from multi variable analysis.

Pearson’s correlation tested the associations between all independent variables, no significant correlation seen. Hosmer and lemeshow test goodness of final model showed the model is fitted (*p* = 0.66, Chi square = 5.90). In multivariable logistic regression analysis, mothers’ educational level, fathers’ education level, house hold income, age of children, dietary diversity score, meal frequency and had nutrition counseling on child feeding were factors significantly associated with adequate vitamin A rich food consumption among children aged 6–59 moths (Table 3).

**TABLE 3:** Bivariable and multivariable binary logistic regression result for factors associated with vitamin A consumption among children age 6–59 months North Shoa Zone, Oromia, Ethiopia, 2022.

Variables	Vitamin A consumption		COR (95%, CI)	AOR (95%, CI)	*P*-value
	Adequate	Not adequate			
**Education of mother**					
No formal education	338	194	Ref	Ref	**–**
Primary school	159	91	0.99 (0.73, 1.36)	1.03 (0.74, 1.42)	0.89
Secondary school and above	73	61	**2.09 (1.42, 3.06)**	**1.81 (1.19, 2.75)**	**0.005**
**Father education**					
No formal education	293	156	Ref	Ref	**–**
Primary school	166	116	1.31 (0.96, 1.78)	1.23 (0.89, 1.70)	0.19
Secondary school and above	99	86	**1.62 (1.15, 2.31)**	**1.54 (1.05, 2.23)**	**0.03**
**Family size**					
< 5	203	152	**1.28 (1.12, 1.46)**	1.27 (0.58, 1.95)	0.07
> 5	355	206	Ref	Ref	–
**Father occupation**					
Income/month	–	–	–	–	–
Less than 2,000	157	119	Ref	Ref	–
2,500–3,499	225	101	1.03 (0.74, 1.43)	0.63 (0.45, 0.89)	0.56
3,500 and above	176	138	**2.78 (1.12, 4.42)**	**2.18 (1.24, 3.82)**	**0.009**
**Child age in month**					
6–11	50	37	Ref	Ref	–
12–17	44	62	**0.57 (0.35, 0.92)**	**0.56 (0.33, 0.90)**	**0.016**
18–23	32	68	**0.45 (0.28, 0.73)**	**0.46 (0.29, 0.74)**	**0.001**
24–29	52	101	**0.40 (0.27, 0.63)**	0.44 (0.29, 2.74)	0.12
30–35	73	97	0.39 (0.25, 1.59)	0.42 (0.27, 1.65)	0.15
36–41	48	95	0.31 (0.23, 1.45)	0.30 (0.32, 1.45)	0.20
42–59	59	98	0.29 (0.20, 1.32)	0.23 (0.12, 1.18)	0.31
**Nutrition counseling**					
Yes	327	185	Ref	Ref	**–**
No	231	173	**1.32 (1.01, 1.73)**	**1.31 (1.10, 1.73)**	**0.04**
**Discussed on child feed**					
Yes	134	120	Ref	Ref	–
No	424	238	**1.60 (1.19, 2.14)**	1.43 (1.00, 1.86)	0.05
**Knowledge of vt A**					
Good	116	72	**1.67 (1.05, 2.26)**	1.16 (0.38, 1.91)	0.06
Poor	442	286	Ref	–	–
**Dietary diversity**					
< 5	467	291	Ref	Ref	–
≥ 5	91	67	**1.77 (1.29, 2.43)**	**1.48 (1.05, 2.07)**	**0.02**
**Meal frequency**					
< 4	312	169	Ref	Ref	–
≥ 4	246	189	**1.41 (1.09, 1.85)**	**1.37 (1.04, 1.81)**	**0.03**

Bold values indicate statistically significant variables odd ratio (OR), confidence interval (CI), and *p*-value meeting the threshold for significance (typically *p* < 0.05).

Accordingly, the odds of consumption of vitamin A among children 6–59 months aged were 1.81 times higher among children whose mothers had secondary and above educational level (AOR = 1.81, 95% CI: 1.19, 2.75) as compared to with mothers had no formal education. Likewise, adequate vitamin A consumption among children aged 6–59 months old were 1.54 times higher among children whose fathers’ had fvary and above educational level (AOR = 1.54, 95% CI: 1.05, 2.23) as compared to those children their fathers’ had no formal education.

As the age of child increases, the likelihood of consuming food groups rich by vitamin A decreases. Being in between age group 12–17 months decrease consumption of vitamin rich food by 44% 0.56 (0.33, 0.90), and being in between age group 18–23 months deceases adequate consumption of vitamin A rich food by 54% 0.46 (0.29, 0.74) as compared to age group in between 6 and 11 months.

Regarding dietary, adequate vitamin A rich food consumption among children aged 6–59 months old 1.48 times higher among children those had dietary diversity five and above per day (AOR = 1.48, 95% CI: 1.05, 2.07) as compared to those had less than five dietary diversity. Likewise, odds of consumption of vitamin A among children aged 6–59 months were 1.37 times higher among children had meal frequency four and above per day (AOR = 1.37, 95% CI: 1.04, 1.81) as compared to those had below. Children of mothers those had nutrition counseling on child feeding were 1.31 more likely consume vitamin A rich food (AOR = 1.31, 95% CI: 1.10, 1.73) as compared to children those mother had no counseling. The odds of consumption of vitamin A rich food among children aged 6–59 months from household those had 3,500 and above ET birr income/month were 2.18 times more likely to consume adequate vitamin A rich food (AOR = 2.18, 95% CI: 1.24, 3.82) as compared to house hold had less than 2,000 ET birr/month.

## Discussion

The study found that 39.1% of children aged 6–59 months consumed adequate vitamin A-rich foods in the week prior to the survey. This result aligns with findings from similar studies conducted in Ethiopia (14, 24), indicating a consistent pattern of suboptimal vitamin A consumption among young children in the country. But, the study’s result was higher than that of the Kachabila district in southern Ethiopia, which reported only 28.8% of children had consumed vitamin A-rich foods within 24 h of the survey (25). This discrepancy may be due to different dietary recall periods. Our study used a 1 week recall, while the Kachabila study used 24 h recall, potentially underestimating vitamin A intake. A longer recall period may better reflect regular dietary patterns, explaining the higher percentage in our study.

However, the results of this study is lower than retrospective study in LMICs children consume foods rich in vitamin A was 55% (26). A possible reason for the discrepancy may be due to a review of LMICs that aggregated the proportions from different countries who received vitamin A-enriched foods. From the review, Ethiopia had the lowest share of children receiving vitamin-A-rich foods 26% (26). Overall, the relatively low percentage of children consuming adequate vitamin A-rich foods suggests that more efforts are needed to improve dietary diversity and access to vitamin A-rich foods in Ethiopia.

The study found that less than 4% of children aged 6–59 months consumed 3–4 servings of fruits and vegetables per day, and only 2% of children met the WHO recommendation of five servings per day. These findings suggest that the consumption of fruits and vegetables is significantly below recommended levels in the study area.

One possible explanation for this low consumption could be food insecurity, which is prevalent in some parts of North Shoa. Food insecurity often limits the availability and access to diverse and nutritious foods, including fruits and vegetables. In regions experiencing food scarcity, families may prioritize staple foods such as grains, which are often less nutrient-dense compared to fruits and vegetables.

Additionally, the timing of the data collection, which occurred during the high-risk dry season, may have contributed to the observed low intake. During this period, the availability of fresh fruits and vegetables typically declines, making it more difficult for households to access these foods. Another contributing factor may be the lack of irrigation systems in the area, which restricts the ability to grow fruits and vegetables year-round.

Regarding factors, maternal educational level, parental educational level, house hold income, age of children, dietary diversity, meal frequency and mothers’ had nutrition counseling on child feeding were factors significantly associated with consumption of vitamin A among children aged 6–59 months.

Children of mothers with secondary or higher education were 1.81 times more likely to consume adequate vitamin A-rich foods than those have no formal education. This finding is consistent with studies conducted in Ethiopia (24), and in India (27). Women with secondary or higher education tend to have better skills in accessing healthcare services, which may include guidance on child nutrition, dietary diversity, and the prevention of vitamin deficiencies. Educated mothers are also more likely to be aware of the importance of dietary diversity and to have the resources and decision-making power to improve their household’s nutrition, including making informed food choices (28).

Similar to the influence of maternal education, children of fathers with secondary or higher education were 1.54 times more likely to consume adequate vitamin A-rich foods than those have no formal education. Educated fathers are more likely to be aware of the nutritional needs of children and the importance of vitamin A for health. They may also be better informed about child care practices and the long-term benefits of providing a diverse and nutrient-rich diet. Education can enhance the ability of fathers to make informed decisions regarding food selection and meal preparation, which ultimately benefits their children’s nutrition (29).

The study found that as the age of the child increases, the likelihood of consuming vitamin A-rich foods decreases. These findings are consistent with other studies from Ethiopia (14, 25, 30), which have also shown that as children grow older, they are less likely to consume vitamin A-rich foods. As children grow older, they typically begin eating a more varied diet, which may lead to a lower focus on nutrient-dense foods such as fruits and vegetables that are rich in vitamin A. Moreover, as children start to consume a wider range of foods, their diet may become less regulated, and the intake of specific nutrients, such as vitamin A, may not be as carefully monitored.

Children aged 6–59 months from households earning ≥ 3,500 ETB/month were 2.18 times more likely to consume adequate vitamin A-rich foods than those from households earning < 2,000 ETB/month. This finding aligns with previous study in Ethiopia, with poorer households being less likely to consume vitamin A rich foods (14). A study in Nepal found that children from the poorest wealth quintile had lower odds of consuming a diverse diet, including vitamin A-rich foods like legumes, nuts, dairy, and flesh foods. This suggests wealthier households are better able to afford a variety of nutrient-dense foods. Additionally, children from wealthier families tend to eat more frequently (e.g., three or more meals a day), which may lead to better nutritional outcomes.

Children aged 6–59 months consuming five or more food groups daily were 1.48 times more likely to consume adequate vitamin A-rich foods than those with lower dietary diversity. This result is consistent with studies from the Tigray region (31), Amhara region (32), Burkina Faso (33), and Kenya (34), all of which have shown that greater dietary diversity is associated with higher consumption of nutrient-rich foods, including those rich in vitamin A. The positive relationship between dietary diversity and vitamin A consumption may be due to the fact that a more diverse diet increases the likelihood of consuming a wide range of foods, including those rich in essential vitamins and minerals.

Children aged 6–59 months who ate four or more meals per day were 1.37 times more likely to consume adequate vitamin A-rich foods than those with fewer meals. This finding is consistent with studies conducted in Ethiopia (35) and Malawi (35) which also found that higher meal frequency is associated with better consumption of nutrient-rich foods, including those rich in vitamin A. Increased meal frequency often leads to a greater opportunity to introduce a variety of foods into the child’s diet.

Consumption of vitamin A among children aged 6–59 months were 1.31 times higher among children’s mother those had nutrition counseling as compared to had no nutrition counseling. This finding is consistent with studies from Ethiopia (25) and Nigeria (36). The positive association between nutrition counseling and vitamin A consumption may be attributed to the fact that mothers who receive counseling are more likely to have greater knowledge about the importance of specific nutrients, such as vitamin A, and the types of foods that provide them.

### Strength of the study

Uses of large sample size with community based study represent target population help to promote program intervention related to improved vitamin A-rich food consumption within these communities.

### Limitation of the study

The data was collected questionnaire based on the respondent memory, so, the study may be prone to both recall bias and social desirability. To minimize these biases, we employed several strategies during data collection: Multiple/prone/Questions: We incorporated multiple questions about the same food item, asked in different ways, to cross-check responses and improve accuracy. This method helps reduce the risk of over- or underreporting due to memory recall. Trained Data Collectors: Our data collection was conducted by trained enumerators who were familiar with the local context and culture. They were trained to encourage accurate responses and reduce social desirability bias by creating a comfortable and non-judgmental environment during interviews. Confidentiality Assurance: To minimize social desirability bias, we emphasized the confidentiality of responses, assuring participants that their answers would not be shared. Seasonal variation in dietary consumption might be another limitation since our study was conducted during the dry season.

## Conclusion and recommendation

In general, this study found that the consumption of vitamin A rich food among children aged 6–59 months old was low in North Shewa Zone, Oromia, Ethiopia. Secondary and above educational levels of child parents, age of children in between 12–17 and 18–23 months, house hold income/month 2500–3500ET birr, five and above dietary diversity per day, four and above meal frequency per day and having discussion on child feeding were factors increase the consumption of vitamin A rich food among children in study area.

It is recommended that relevant health authorities at the local, regional, and national levels use the findings from this study to set priorities and target interventions aimed at promoting the consumption of vitamin A-rich foods among children aged 6–59 months, focusing on food-based approaches to reduce micronutrient deficiencies.

It is recommended that relevant authorities at the local, regional, and national levels use should consider addressing household income disparities by incorporating strategies that promote income generation and economic growth, thereby improving access to vitamin A-rich foods.

It is recommended that relevant educational authorities at the local, regional, and national levels could strengthen the educational level of parents, particularly aiming to increase the percentage of individuals with at least secondary education. Adult education initiatives should also be promoted to enhance overall community knowledge, particularly around nutrition and health.

Healthcare providers should focus on strengthening nutrition counseling regarding dietary diversity and meal frequency for women with children under five, while also addressing the nutritional needs of children as they grow older.

For researchers, it would be valuable to conduct further year-round assessments to understand the seasonal variations in the consumption of vitamin A-rich foods, allowing for more accurate data and tailored interventions.

## Data Availability

The raw data supporting the conclusions of this article will be made available by the authors, without undue reservation.
