# Supplementary material for: Consumption of vitamin A rich foods and its associated factors among children aged 6–59 months in North Shoa Zone, Oromia regional state, Ethiopia
Source: Front Nutr. 2025 Jun 16;12:1526292. doi: 10.3389/fnut.2025.1526292 (PMC12206797; doi:10.3389/fnut.2025.1526292)
Supplement: Supplementary file 1 [file Table_1.docx]

**Data collection tools**

English version questionnaires on consumption of vitamin A source foods and associated factors among children aged 6-59 months in North Shoa Zone, Oromia, Ethiopia, 2021/22

General information

1. Ethiopian calendar date of interview _____/______

2. Name of the interviewer:______________________ _____signature __________

3. Name of supervisor _____________________________ signature ___________

3. Location: Woreda_____________ Kebele___________1. Urban 2. Rural

Table1: Data collection tools

| **Part 1: Socio demographic and economic information for mother/caregivers and children** | | | | | | |
| --- | --- | --- | --- | --- | --- | --- |
| S.NO. | Questions | | Responses | | | Skip |
| 100 | How old are you? | | …………………...years (completed age) | | |  |
| 101 | What is your Religion? | | 1. Orthodox 2. Protestant 3. Muslim 4. Other (specify) __________ | | |  |
| 102 | What is your ethnicity? | | 1. Oromo 2. Amhara 3. Other(specify) | | |  |
| 103 | What is your Marital status? | | 1. Single 2. Married 3. Divorce 4. Widowed 5. Others --------- | | |  |
| 105 | What is your Education level? | | 1. Unable to read and write  2. Able to Read and write  3. Primary education  4. Secondary education  5. Diploma  6. degree and above | | |  |
| 107 | What is your occupation? | | 1. Farmer/Agricultural worker 2. Daily laborer  3.Trader  4. Employee  5.House wife  6.other (specify) _____________ | | |  |
| 108 | Husband /House head educational status? | | 1. Unable to read and write  2. Able to Read and write  3. Primary education  4. Secondary education  5. Diploma  6. degree and above | | |  |
| 109 | How many Family members are there in your Home? | | ______________ | | |  |
| 110 | How much is your monthly Income of the household? (in Eth. Birr)? | | 1 less than 2000  2.2000-2500  3. 3000 and above brr | | |  |
| **Child related Questionnaires** | | | | | | |
| 111 | How many children age 6-59 months do you have /are found in your house? | | Randomly select one child from children 6—59 months age | | |  |
| 112 | What is the sex of child? | | 1. Male  2.Female | | |  |
| 113 | What is the age of child? a calendar of local events may use to determine the age of the child as accurately as possible. | | (in months) _______________ | | |  |
| 114 | For how long you feed breast milk | | 1less than 6 month  2. 6 month and greater | | |  |
| 115 |  | |  | | |  |
| **Part 2: Health and nutritional related variables** | | | | | | |
| 201 | Did you visit health facility for ANC during your pregnancy for this child? | | 1. Yes 2. No | | |  |
| 202 | Where did you gave birth to this child /Place of delivery/? | | 1.Home  2.Hospital  3. Health Center  4.Other (specify)---- | | |  |
| 203 | Have you received post natal care? | | 1. Yes 2.No | | |  |
| 204 | Have you received nutritional counseling to give your child fruits and vegetables | | 1.Yes  2.No | | |  |
| 205 | If you say yes, what is the primary source of information? | | 1.Health Professionals  2.Health extension workers  3.Friends  4. Neighbors  5.Radio  6.TV  7.Other (specify) | | |  |
| 206 | Have you received nutritional counseling to give your child animal source foods | | 1.Yes  2.No | | |  |
| 207 | If you say yes, what is the primary source of information? | | 1.Health Professionals  2.Health extension workers  3.Friends  4. Neighbors  5.mass media  7.Other (specify) | | |  |
| 208 | If your answer is mass media, which one? | | 1. Radio 2. Television 3.Newspaper 4.Internet access  5.other specify | | |  |
| 209 | How many times did you watch TV /week | | 1. watch TV-------- per week  2. List to radio ----per week  3. Read newspaper---- perweek  4.other | | |  |
| **Part:3 The Helen Keller International Food Frequency Questionnaire** | | | | | | |
| 300 | | How many days, in past seven days, did your child eat specific food items listed below | | Number of days eaten per week |  | |
| 301 | | Milk /whole | |  |  | |
| 302 | | Meat(beef,lamb,chiken ) | |  |  | |
| 303 | | Organ meat (Liver, kidney, heart) | |  |  | |
| 304 | | Fish | |  |  | |
| 305 | | Egg | |  |  | |
| 307 | | Butter | |  |  | |
| 309 | | Hot peppers | |  |  | |
| 310 | | DGLVs(spinach, kale ,etc |  | |  | |
| 311 | | Carrots |  | |  | |
| 312 | | Mango |  | |  | |
| 313 | | Pumpkin |  | |  | |
| 314 | | Papaya |  | |  | |
| 315 | | Sweet potato |  | |  | |
| 316 | | Lentil or others legumes |  | |  | |
| 317 | | Vitamin A fortified foods |  | |  | |
| 318 | | Has (child’s name) ever received vitamin A capsule/drops like this one?(show capsule )  information may available from card and/or book. | 1. Yes 2. No if the answer is No skip next Question | |  | |
| 319 | | If the answer is yes they, “Did (child’s name) receive a vitamin A drop within the last six months?” | 1. Yes  2. No | |  | |
| 320 | | If yes, did he/she consume a vitamin A drop in last 12 months | 1. Yes  2. No | |  | |
|  | |  |  | |  | |
| **Part4: mother/caregiver knowledge on vitamin A rich foods( analysis know/don’t know)** | | | | | | |
| 400 | | Have you heard about vitamin A deficiency or lack of vitamin A? | 1. Yes 2. No 3. Don’t know/ no answer | | |  |
| 401 | | If yes :can you tell me how you can recognize someone who lacks vitamin A in his/ her body | 1. Weakness /feels less energetic 2. Be more likely to be sick(less immunity to infection ) 3. Other 4. Don’t know | | |  |
| 402 | | What causes lack of vitamin A in the body | 1. Poor variety of foods 2. Eat too little food /not eat much/(poor intake) 3. Other 4. Don’t know | | |  |
| 403 | | How can one prevent a lack of vitamin A in the body | 1. Feed vitamin A rich foods 2. Eat food fortified by vitamin A 3. Give/take vitamin A supplementation 4. Others 5. Don’t know | | |  |
| 404 | | Can you list animal source of vitamin A ? | 1. Liver 2. Kidney 3. Heart 4. Egg 5. Milk 6. Other 7. Don’t know | | |  |
| 405 | | Can you list vegetable source of vitamin A? | 1. Sweet potato 2. Carrot 3. Pumpkin 4. Squash 5. Other 6. Don’t know | | |  |
| 406 | | Can you list fruits source of vitamin A ? | 1. Ripe Papaya 2. Ripe Mango 3. Apricot 4. Dried peach 5. Palm iol 6. Other 7. Don’t know | | |  |
| 407 | | Can you list vitamin A fortified foods? | 1. Oil 2. Fats 3. Sugar 4. Infant formula 5. Other 6. Don’t know | | |  |
| **Practice questionnaires , I would like to ask you about particular foods you may eat on their own or as part of dish** | | | | | | |
| 408 | | Yesterday ,during the day and night ,did you eat any of the following animal foods? | Liver 1. Yes 2.no  Kidney 1. Yes 2. No  Heart 1. Yes 2.no  Eggs 1. Yes 2. No  Milk/cheese /yougort or other dairy products 1. Yes 2. No | | |  |
| 409 | | Yesterday, during the day and night ,did you eat any of the following vegetable foods? | Sweet potato 1. Yes 2. No  Carrot 1. Yes 2. No  Pumpkin 1. Yes 2. No  Squash 1. Yes 2. No | | |  |
| 410 | | Yesterday ,during the day and night ,did you eat any of the following green leafy vegetable foods | Amaranths 1. Yes 2. No  Spanish 1. Yes 2. No  Other dark green leafy vegetable  1. Yes 2. No | | |  |
| 411 | | Yesterday ,during the day and night ,did you eat any of the following fruits | Ripe Papaya 1. Yes 2. No  Ripe Mango 1. Yes 2. No  Apricot 1. Yes 2. No  Dried peach 1. Yes 2. No  Palm oil 1. Yes 2. No  Red palm oil 1. Yes 2. No | | |  |
| **Attitudes questionnaires towards consumption of vitamin A rich foods** | | | | | | |
| 412 | | How likely do you think your child is to lack vitamin A in his/her body? | 1. Not likely 2. you are not sure 3. Likely | | |  |
| 413 | | How serious do you think a lack of vitamin A is ? | 1. Not serious 2. You are not sure 3. Serious | | |  |
| 414 | | How good you think it is to prepare meals with vitamin A rich foods such as carrots green leafy vegetables ,sweet potato or liver ? | 1. Not good 2. You are not sure 3. Good | | |  |
| 415 | | How difficult it is for you to prepare meals with vitamin A rich foods | 1. Not difficult 2. You are not sure 3. Difficult | | |  |
| 416 | | How confident do feel in preparing meals with vitamin A rich foods | 1. Not confident 2. You are not sure 3. Confident | | |  |
| 417 | | How much do you like the test of vitamin A rich foods (milk,liver,…) | 1. Dislike 2. Neutral 3. Like | | |  |

Dietary diversity

I would like to ask you about the types of foods that your child took from sunrise yesterday to sunrise today with in past (24) hour. Read the list of foods below and place a one in the box if child ate the food in question, place a zero in the box if a child did not.

| **No** | **Food group** | **Examples** | **1-Yes**  **0- No** | **Over 24hr** |
| --- | --- | --- | --- | --- |
| 401 | grain ,white roots & tubers | Maize, teff, rice, wheat, sorghum, or any other grains or foods made from these (e.g. Injera, bread, noodles, pasta ,macaroni, kinche, rice, atmit, porridge ,bread, local pasty or other grain products),& White potatoes, sweet potato, carrot, or other foods made from roots like godore, enset |  |  |
| 402 | Vitamin A rich fruits, vegetables & tuber | ripe mango, apricot (fresh or dried), ripe papaya, dried peach, and fruit juice made from these, carrot, or sweet potato that are orange inside & other locally available vitamin A rich vegetables (e.g. red sweet pepper) |  |  |
| 403 | Other fruits and vegetables | Other vegetables (e.g. tomato, onion, eggplant &other locally available vegetables other fruits, including wild fruits. |  |  |
| 404 | Meat, poultry , fish and sea food | liver, kidney, heart or other organ meats or blood-based foods beef, pork, lamb, goat, chicken ,canned meat, Fresh or dried fish |  |  |
| 405 | Eggs | Eggs |  |  |
| 406 | Legumes, nuts and seeds | Dried beans, dried peas Beans, lentils, nuts, seeds or foods made from these ( eg. shiro wet, kik wet, misir wet, shimbra kolo, bakela ashuk, boloke……) |  |  |
| 407 | Milk and milk products Milk | Milk, cheese, yogurt or other milk products like aguat, arera… formula milk,cheese cream |  |  |
| 408 | Fruit and vegetable vitamin A rich food |  |  |  |
| 409 | Other fruit and vegetable |  |  |  |
| 410 | Oil and fat |  |  |  |

7.1 Guca waliigaltee Guyyaaa_____/_____/____

Akkam bultan/ooltan? Maqaan koo _______________________________________. Ani bakka _________jedhamu hojjechaa jira . Nut qoranno waa’ee nyaata vitaminii A badhadhan fi kannenen isaanin walqaban irratti asuma zonii shawaa kaabaa,oromiyaa ,itoopiyaa keessat hojechaa jirra. Odeeffannon nut funaannu kun mootummaa karoora tajaalila fayyaa fi nyaata akka baasu gargaara .ati daa’ima kee waliin qorannoo kaaf fimatamtaniittu. Qaaffiileen daqiiiqa 20 fudhachuu dandaha.The questions usually. Want isin deebisan hunduu iccittin qabama namittu dabarsamee hin kennamu. Qorannoo kana keessatti hirmaachuu keessaniif miidhan isi irra gahu tokkollee hin jiru.yaadni keessan qorannoo kanaaf baayyee nu barbaachisa garuu dirqama hin qabda .

Gaaffii qabdu?

Gaaffii keenya itti yaa fufnuu ? 1. eeyyee 2. lakki

Yoo eeyee tahe, gaaffii itti fufi .

Yoo lakkii tahe,galateeffadhuutii nama biraatti darbi

Qorannoo kana irratti gaaffii adda/dabalataa yoo qabaattan warren kanaan ganii kamuu qunamuu dandeessu.I

No. Name Phone number Email address Address

1 Mrs. Meseret Moroda ( PI) 0967711171 [meseret2022@gmail.com](mailto:meseret2022@gmail.com)

2. Mr. Girma Garedew (CI) 0913937731 [girmaggg73@gmail.com](mailto:girmaggg73@gmail.com)

3. Mr. Rabik shukure (CI) 0967659476 [rebikshukure92@gmail.com](mailto:rebikshukure92@gmail.com)

9.3 Gaaffilee afaan oromootin

Meeshaalee oddeffannoo ittin funaanamu

Bara 2014 godina shawaa kaabaa,naannoo Oromia ,biyya ,Itoopiyaa keessatti ijoollee ji;a 6-23 jiraniif nyaata madda vitaminii A qaban fi kanneen kanaan walqabatan uunka afaan oromootiin.

Oodeeffannoo waliigalaa

1. Guyyaa gaaffii akka lakkoofsa Itiopiyaattii______/___________/__________
2. Maqaa nama ragaa funaanuu ________________________
3. Iddoo ragaan funaanamu aanaa_________qabalee________1. magaala 2. baadiyyaa

| KutaI:Gaafii haala hawaasummaa fi dinagdee kan agarsiisan haadhaaf /guddistuuf | | | | | | | | | | | | | | | | | |
| --- | --- | --- | --- | --- | --- | --- | --- | --- | --- | --- | --- | --- | --- | --- | --- | --- | --- |
| T.L | | Gaaffilee | | | Deebiiwwan | | | | | | | Irra utaaluu | | | | | |
| 100 | | Irra caalatti daa’ima kan eenyutu kunuunsa? | | | 1.haadha 2.akkayoo 3.oboleettii  4.guddistuu 5. kan biraa ibsa  Kano lii keessa kan yeroo baayye daa’ima kan kununsuu filadhu gaaffile kannenn armaan gadii gaafadhu | | | | | | |  | | | | | |
| 101 | | Umuriin keessan meeqa? | | | …………waggaan | | | | | | |  | | | | | |
| 103 | | Amantiin keessan maalii? | | | 1. ortodoksii 2. pirotestaantii 3. musliima  4.kan biraa(ibsaa) __________ | | | | | | |  | | | | | |
| 104 | | Sabnikee maaliidha? | | | 1. oromoo 2. Amaraa 3.kan biraa (ibsa) | | | | | | |  | | | | | |
| 105 | | Haala fuudhaa fi heerumaa yeroo ammatti? | | | 1.kanhin heerumne 2. kan heerumte  3. kan walihikte 4. kan abban mana du’e  5. kan biraa ---- | | | | | | |  | | | | | |
| 106 | | Sadarkaan barnoota kee maaliidha? | | | 1. Bareessuu fi dubbisuu hin dandeessu 2. Bareessuu fi dubbisuu dandeessu 3. Sadarkaa tokkoffaa (kutaa 1-8) 4. Sadarkaa lammaffaa (Kutaa 9-12) 5. Seertifiikeetii 6. Diploomaa 7. Digrii fi isaa olii | | | | | | |  | | | | | |
| 107 | | Hojiinkee/haadha daa’ima maalii | | | 1.Haadha mana 2. Hojjata mootummaa  3. Mit-mootummaa 4.kan biraa (ibsi) | | | | | | |  | | | | | |
| 108 | | Sadarkaan barnoota abba daa’ima maaliidha? | | | 1. Bareessuu fi dubbisuu hin dandeenye 2. Bareessuu fi dubbisuu kan danda’u 3. Sadarkaa tokkoffaa (kutaa 1-8) 4. Sadarkaa lammaffaa (Kutaa 9-12) 5. Seertifiikeetii 6. Diploomaa 7. Digrii fi isaa olii | | | | | | |  | | | | | |
| 109 | | Hojiin abbaa maalii ? | | | 1.Qotee bulaa 2. Hojjata mootummaa  3.Mit-mootummaa 4. daldalaa  5. Kan biro(yoo jirate adda basi) | | | | | | |  | | | | | |
| 110 | | Mana keessan keessa nama meeqa jiraata | | | ______________lakkofasan | | | | | | |  | | | | | |
| 111 | | Galiin maatii ji’aan meeqa | | | ________________birr Itoopiyaan | | | | | | |  | | | | | |
| Gaaffilee daa’iman walqabatan | | | | | | | | | | | | | | | | | |
| 201 | | | Daa’imman ji’a 6-23 gidduutti argaman meeqa qabdan ? | | | | | Daa’imman ji’a6-23 jiran keessa tokko haala waliqixaan taheen /lotoriin filadhu | | | | |  | | | | |
| 202 | | | Saala daa’ima maaliidha? | | | | | 1. dhiira 2. durba | | | | |  | | | | |
| 203 | | | Umuriin daa’ima meeqa ? | | | | | ____________ ji’aan | | | | |  | | | | |
| 204 | | | Daa’ima (maqaa waamii) harma hoosistaa jirta | | | | | 1. eeyyee 2. lakkii | | | | | 2 =>301 | | | | |
| 205 | | | Deebiin eeyyee yoo tahe, kaleessa ganamaa ykn galgala harma hosiste jirtaa? | | | | | 1.eeyyeee 2.lakkii | | | | |  | | | | |
| 206 | | | Erga desse booda hangamitti harma hoosista | | | | | ____ __ sa’atti/guyyaan | | | | |  | | | | |
| 207 | | | Hanga ji’a meeqatti harma qofaa hosista/e? | | | | | _______ji’a | | | | |  | | | | |
| 208 | | | Ji’a ja’a gaditti harma irratti waa dabaltuu jira? | | | | | 1. eeyyee 2. lakkii | | | | |  | | | | |
| 209 | | | Hanga ammaa harma hosisaa jirtaa? | | | | | 1. eeyyee 2. Lakkii | | | | |  | | | | |
| Kutaa 2 ፡ Gaaffilee tajajila fayyaa fi nyaata waliin kan walqaban | | | | | | | | | | | | | | | | | |
| 300 | Tilmaaman manni yaalaa dhihoo keessa jiru kiloometra hangam isin irraa faggata? | | | | | | | | __________KM | | | | | | |  | |
| 301 | Daa’ima kanaaf tajaajila ulfaaf mana yaalaa deemaa turte? | | | | | | | | 1. eeyyee 2.lakkii | | | | | | |  | |
| 302 | Daa’ima kana eessatti deesse ? | | | | | | | | 1. manatti 2.mana yaalaa | | | | | | |  | |
| 303 | Erga deesse booda tajaala da’umsa boodaaf gara mana yaalaa deemte? | | | | | | | | 1.Eeyyee 2. lakkii | | | | | | |  | |
| 304 | Gorsa /odeeffanno haala daa’ima fi ijoollee xixiqqoo itti nyaachisan (IYCF) argatte beekta? | | | | | | | | 1.Eeyyee 2. lakkii | | | | | | | 2 =>309 | |
| 305 | Haala qabatamaan nyaata daa’ima fi ijoollee xixxiqqoo akkamitti akka qopheessanii fi nyaachisan hoo? | | | | | | | | 1.Eeyyee 2. lakkii | | | | | | |  | |
| 306 | Deebiin kee eeyyee yoo tahe, madda odeefannoo kee isa guddan eenyudha? | | | | | | | | 1. ogeessota fayyaa  2.hiriyoota/garee dubartoota  3 mastaritii hawasaa (tv,radio,.  4.kan biraa( ibsaa) | | | | | | |  | |
| 307 | Deebiin kee mastaritii hawasaa (መገናኛ ብዙኃን) yoo tahe ,isa kami? | | | | | | | | 1. radio  2. television  3. gaazexaa  4. tajaajia interneeta  5.kan bira(ibsaa) | | | | | | |  | |
| 308 | Torbeetti mass media yeroo meeqa ilaaltu/dhageeffattu | | | | | | | | 1. telvejiin ilalauu _______  2. radio dhageeffachuu ___  3. gazexaa dubbisuu _____  4.kan biraa | | | | | | |  | |
| 309 | waa’ee nyaata daa’imanii fi ijoollee xixiqqoo kan irratti mar’iattanii beektu | | | | | | | | 1.eeyyee 2. lakkii | | | | | | | 2=>311 | |
| 310 | deebiin kee eeyyee yoo tahe ,Eenyu wajjin? | | | | | | | | 1. Miseensa maatii 2. Garee dubartoota 3. Ogeessota fayyaa/ekisteenshinii | | | | | | |  | |
| 311 | Daa’ima( maqaa waami) harma hoosisuun alatti nyaata kamuu jalqabsistanii? | | | | | | | | 1.Eeyyee 2.Lakkii | | | | | 2=>401 | | | |
| 313 | Deebin eeye yoo tahe, harmaan alatti kaleessa nyaata kamuu nyaate ture? | | | | | | | | 1. Eeyee 2. Lakkii | | | | |  | | | |
| 312 | Umurii/ji’a meeqa irratti nyaata dabalataa harmaan alattii jalqabsiistan | | | | | | | | ____________ji’aan | | | | |  | | | |
| **Gaaffilee Nyaata gosa adda addaa nyaachuun walqabatan ፡Amma (maqaa daa’imaa) kaleessa guyyaa fi halkan (sa’aatii 24 darbe keessatti) mana keessattis tahe alatti nyaate isin gaafadha ?** | | | | | | | | | | | | | | | | | |
|  | Gartuu nyat | | | Akaakuuu nyaataa | | | | | | | Deebii | | | | | |  |
| 401 | Midhaan sanyii,hundee fi jirma isaanii nyataman | | | Boqqolloo,xaafii,ruuzii qamadii,aajjaa ,misingaa ykn nyaata kannen irraa hojjetame buddeena, daabboo/qixxaa/ bassoo qincee, paastaa, mokoranii bulluqa, marqaa. Dinnicha adii, kaarotii, dinnicha mi’awaa, duubbaa fi nyyata kanneen irraa hojjetame | | | | | | | 1.eyee 2.lakki  3.hin beeku | | | | | |  |
| 402 | Midhaan dheedhii | | | Baaqelaa,atara boloqqee ,misira,lawuzii,ykn nyaata kanneen irraa hojjetame fkn waxii shiroo ,kikkii ,misiraa ,akaayii shumburaa ,mulluu baaqelaa ykn boloqqee | | | | | | | 1.eeye  2.lakki  3.hin beeku | | | | | |  |
| 403 | Foon fi gosa isaa | | | Tiruu,kalee,onnee,ykn foon qaama kan biro ykn nyaata foon (qotiyyoo,hoolaa re’ee,hindaqqoo qurxummii..) irraa hojjetaman | | | | | | | 1.eeye  2.lakki  3.hin beeku | | | | | |  |
| 404 | Aannanii fi gosa isaa | | | Aannan, ayibii ,itittuu ykn bu’aa aannanii kan akka aareera | | | | | | | 1.eeye  2.lakki  3.hin beeku | | | | | |  |
| 405 | Killee | | | Killee | | | | | | | 1.eeye 2.lakki  3. hin beeku | | | | | |  |
| 406 | mudulaale fi ,kudhulaalee vitaminii A qaban | | | Goomman ,qoosxaa ,paappayyaa,mango ykn duubbaa juusii,kaarotii,miximixxaa ,dinicha mi’awaa kannen kessi isaanii burtukaana fakkatan | | | | | | | 1.eeye  2.lakki  3.hin beeku | | | | | |  |
| 407 | Muduraalee fikudhuraale Kanneen biro | | | Muduraalee kannen biro fkn,timaatimii ,shunkurtii hundee diimaa fi kan biro kudhuraalee bosonaa dabalatee | | | | | | | 1.eeye  2.lakki  3.hin beeku | | | | | |  |
| 408 | Nyaata  mi’awaa | | | chakoleeta,kekii,buskuta,chipsii,bomboliinoo,qoqorii karameella, | | | | | | | 1.eeye  2.lakki  3.hin beeku | | | | | |  |
| 409  410 | Dhugaatii mi’awaa/lallaafaa | | | Miridaa,dhugaatota mi’awaa suqqii irraa bitaman (aannan,juice ) ,Buna ,shahii  **Nyaata biraa yoo jiraate ?**  **asitti barressi__________** | | | | | | | 1.eeye  2. Lakki  3.hin beeku | | | | | |  |
| 411 |  | | | kalessa ganamaa hanga hara ganamaatti [maqaa daa’ima] **si’a meeqa nyaa**ta nyaate?  1.harma kan hodhaniif ________ 2.kan harma hin hooneef _______ | | | | | | |  | | | | | |  |
|  | **Kutaa፡3 Gaaffilee Helan killerii akka addunyaatti kan deddebitii nyaata agarsisan** | | | | | | | | | | | | | | | | |
|  | Guyyoota turban darban keessatti guyyaa meeqa daa’imni kee (daa’imicha maqaa waamin)nyaata xiqqoo gaditti tarrefaman nyaate ? | | | | | | | | | baayi’na guyyoota nyaata tarrefame nyaate barressi | | | | | |  | |
| 412 | aannan | | | | | | | | |  | | | | | |  | |
|  | Foon | | | | | | | | |  | | | | | |  | |
|  | Tiruu | | | | | | | | |  | | | | | |  | |
|  | Qurxummii | | | | | | | | |  | | | | | |  | |
|  | Killee/killoo kesssa waliin | | | | | | | | |  | | | | | |  | |
|  | Lukkuu/handaqqoo | | | | | | | | |  | | | | | |  | |
|  | Dhadhaa | | | | | | | | |  | | | | | |  | |
|  | Ruuzii /Boqqollo /Ajjaa/Garbuu/Misigaa/Qamadii | | | | | | | | |  | | | | | |  | |
|  | Paastaa, pastinii ,endoomii,mokoronii…kanneen biro | | | | | | | | |  | | | | | |  | |
|  | Ataakiltii baalaa halluu gurracha magariisa qaban (goomman gurraacha,gomman maramaa,…) | | | | | | | | |  | | | | | |  | |
|  | Qoosxaa | | | | | | | | |  | | | | | |  | |
|  | Kaarotii | | | | | | | | |  | | | | | |  | |
|  | Maangoo | | | | | | | | |  | | | | | |  | |
|  | Duubbaa | | | | | | | | |  | | | | | |  | |
|  | Paappayaa(bilchaataa) | | | | | | | | |  | | | | | |  | |
|  | Ocholonii | | | | | | | | |  | | | | | |  | |
|  | misira ykn midhaan dheedhii biro | | | | | | | | |  | | | | | |  | |
|  | Timatima | | | | | | | | |  | | | | | |  | |
|  | Dinnicha mi’awaa (sukkar dinnicha) | | | | | | | | |  | | | | | |  | |
|  | Qimamii, fkn masoobilaa ,Mixmixxaa | | | | | | | | |  | | | | | |  | |
|  | Nyaata vitamin A qaban (daakuu aannanii ,faaffaa, zayita qurxummii,dhadhaa atakiltii ) | | | | | | | | |  | | | | | |  | |
|  | Daa’imni kee coba vitamin A fudhateera? (cabsulii itti agarsiisi) | | | | | | | | | 1. eeye 2.lakkii | | | | | | 2=>501 | |
|  | **Ijoollee waggaa tokko oliif** ,deebiin eeyyee yoo tahe daa’imni (maqaa ) ji’a 6 darbeetti keessatti vitamin A fudhatera/tti ? | | | | | | | | | 1. eeye 2. lakkii | | | | | |  | |
|  | Eeyyee yoo tahe ji’a 12 darbe keessatti hoo vitamin A fudhateera ? | | | | | | | | | 1. eeye 2. lakkii | | | | | |  | |
|  | **Kutaa:4 Beekumsa haadha/guddistuu nyaata vitaminii A badhaadhan irratti** | | | | | | | | | | | | | | | | |
| 501 | Waa’ee hanqin vitaminii A dhageesee beekta? | | | | | 1. eeyyee 2. lakkii  3. hin beekan /deebii hin qabu(callisuu) | | | | | | | | | 2=>509 | | |
| 502 | Deebiin G501, Eeyyee yoo tahe nama hirina vitamin A qabu akkamitti adda baasne beekna? | | | | | 1.dadhabbii /miira dadhabbii  2. carraan dukkubaan qabamuu isaanii olka’adha (dandeetti dhukkuba of irraa ittisuu gadibu’uu)  3. kan biraa  4. hin beekan | | | | | | | | |  | | |
| 503 | Hiri’na vitamin A qaama keessattii maaltu fida ? | | | | | 1. nyaata madaalamaa dhabuu  2.nyaata xiqqoo nyaachuu/nyaata gahaa fudhachuu dadhabuu  3. kan biraa 4. hin beekan | | | | | | | | |  | | |
| 504 | Qaama keessatti hir’ina vitamin A akkamitti hambisuu danda’ama? | | | | | 1. nyaata vitamin A badhaadhan fudhachuu /nyaachuu  2. nyaata vitaminii A itti dabalame nyaachuu  3. nyaata dabalata vitaminii A qabu nyaachuu  4. kan biro 5. hin beekan | | | | | | | | |  | | |
| 505 | nyaata bu’aa beelyleedoota vitamin A of keessa qaban tarreessuu dandeechu? | | | | | 1. tiruu 2. Kalee 3. onnee  4. killee 5. Aannan 6. kan biroo  7. hin beekan | | | | | | | | |  | | |
| 506 | Muduraalee vitamin A of keessa qaban tarreessu ni dandeessu? | | | | | 1. dinnicha mi’awaa 2. Kaarotii 3. duubbaa  4. qoosxaa 5. kan biraa 6. hin beekan | | | | | | | | |  | | |
| 507 | Nyaata kudhuraalee vitamimii A of keessaa qaba tarreessu ni dandeessu? | | | | | 1. pappayaa bilchaataa 2.mangoo bilchaataa  3. muuziii 4.burtukaanna 5.aananasii  6.kan biraa 7. hin beekan | | | | | | | | |  | | |
| 508 | Nyaata vitaminii A dabalata qaban tarreessuu ni danadeessu? | | | | | 1. zayitii 2. cooma 3. sukkaara  4. nyaata daa’immanii 5. kan biraa 6. hin beekan | | | | | | | | |  | | |
|  | **Gaaffilee gochaa agarsiisan:Daa’imni keessaniif nyaata kaleessa ganamaa hanga har’a ganamaatti laattaniiif sin gaafachuun barbaada** | | | | | | | | | | | | | | | | |
| 509 | Daa’imni (maqaa daa’ima waami) kaleessa ganamaa hanga har’a ganamaatti kannen armaan gadii bu’aa nyaata beelidootaa ta’an nyaatera? | | | | | | Tiruu 1. Eeye 2. lakki  Kalee 1. Eyee 2.lakki  Onnee 1. Eyee 2.lakki  Killee 1. Eyee 2.lakki  Aannan/itittuu/ 1.Eyee 2. lakki | | | | | | | |  | | |
| 510 | (maqaa daa’ima waami) kaleessa ganamaa hanga har’a ganamaatti mudraalee kannen armaan gadii nyaatera/soorateera ? | | | | | | Sukkara mi’awaa 1. Eeyee 2.lakki  Kaarrotii 1. Eeyee 2.lakki  Duubbaa 1. Eeyee 2.lakki  Kanneen biro 1. Eeyee 2.lakki | | | | | | | |  | | |
| 511 | (maqaa daa’ima waami) kaleessa ganamaa hanga har’a ganamaatti muduraalee baala halluu magariisa gurracha kannen armaan gadii nyaatera/soorateera ? | | | | | | Goomman gurracha 1. Eyee 2. Lakki  Goomman marama 1.Eyee 2.lakki  Qoosxaa 1.eyee 2.lakkii  Kanneen biro 1. Eeyee 2. lakki | | | | | | | |  | | |
| 512 | (maqaa daa’ima waami) kaleessa ganamaa hanga har’a ganamaatti kudhuraalee kannen armaan gadii nyaatera/soorateera ? | | | | | | Papayaa 1. Eeyee 2.lakki  Maangoo 1. Eeyee 2.lakki  Muuzii 1. Eeyee 2.lakki  Burtukaana 1. Eeyee 2.lakki  Kan biro 1. eeyee 2.lakki | | | | | | | |  | | |
|  | **Gaaffilee ilaalcha nyaata vitaminii A badhaadhan soorachuu irratti** | | | | | | | | | | | | | | | | |
| 513 | Daa’imni keessan carraan hanqina vitaminii A qaama isaa/ishii keessatti jiraachuu danda’a jettanii yaaddu ? | | | | | | 1. tahuu hin dandahu  2. hin beeku  3. tahuu dandaha | | | | | | | |  | | |
| 514 | Hanqi’inni vitaminii A hangam yaachisadha jettanii yaaddu? | | | | | | 1. yaachisa/ulfaata miti  2. hin beeku  3. yaachisadha /ulfaatadha | | | | | | | |  | | |
| 515 | Nyaata vitaminii A qaban kannen akka kaarrotii, muduraalee baala hallu gurracha magariisa qaban,sukkar dinnicha ,tiruu , fi kkn qopheessu irratti hangam cimoodha ? | | | | | | 1.gaariidha  2.hin beeku  3.gaarii miti | | | | | | | |  | | |
| 516 | Nyaata vitaminii A qaban qopheessun isiniitti hangam ulfaata? | | | | | | 1. ulfaata mitii  2. hin beeku  3. ulfaatadha | | | | | | | |  | | |
| 517 | Nyaata vitaminii A qaban qopheessuu irratti hangam ofitti amanamumma isinitti dhagahama? | | | | | | 1. ofitti hin amanu  2. hin beeku  3. ofitti amana | | | | | | | |  | | |
| 518 | Nyaata vitaminii A qaban kannen akkan aannan ,tiruu...dhandhama isaa jaalattu ? | | | | | | 1.hin jaaladhu  2. lachuu keessa hin jiru  3. nan jaaladha | | | | | | | |  | | |
